# Crizotinib Associated Renal Cysts [CARCs]: incidence and patterns of evolution

**DOI:** 10.1186/s40644-017-0109-5

**Published:** 2017-02-16

**Authors:** Laird B Cameron, Damian H S Jiang, Kate Moodie, Catherine Mitchell, Benjamin Solomon, Bimal Kumar Parameswaran

**Affiliations:** 1 Department of Medical Oncology, Peter MacCallum Cancer Centre, Grattan Street, Melbourne, Australia; 2 Department of Cancer Imaging, Peter MacCallum Cancer Centre, Grattan Street, Melbourne, Australia; 3 Department of Pathology, Peter MacCallum Cancer Centre, Grattan Street, Melbourne, Australia; 4 Department of Medical Oncology, Peter MacCallum Cancer Centre; Sir Peter MacCallum Department of Oncology, University of Melbourne, Melbourne, Australia

**Keywords:** Crizotinib, Renal cyst, Spontaneous resolution, CT, Non-small cell lung cancer, Anaplastic lymphoma kinase

## Abstract

**Background:**

Novel therapeutic agents recently introduced for the treatment of cancer have several unusual side effects. An increased incidence of renal cystic lesions, often with features concerning for malignancy or infection, has been reported in patients with anaplastic lymphoma kinase (ALK) - rearranged advanced non-small cell lung cancer (NSCLC) treated with Crizotinib. Many of these lesions undergo spontaneous resolution despite developing complex features on imaging. We assess the incidence and patterns of evolution of Crizotinib Associated Renal Cysts [CARCs] at our institute and provide histopathology correlation of their benign nature.

**Methods:**

A retrospective analysis of renal lesions in computerised tomography (CT) scans of 35 patients with advanced *ALK*-rearranged NSCLC who had been prescribed crizotinib at our institution was performed by three radiologists, who analysed the evolution of these lesions, particularly for pre-defined significant and complex changes.

**Results:**

Of 26 patients eligible for this analysis, 4 (15%) had cysts at baseline that remained stable on crizotinib treatment while 11(42%) developed significant change in 28 renal cysts. Commonest pattern of cyst evolution was enlargement from baseline followed by spontaneous regression (17/28 lesions) while other patterns noted were stable lesions, regression from baseline and ongoing enlargement. The median maximum size reached was 23 mm (range 9 – 67 mm) after a median of 178 days (160 to 1342) on crizotinib. Complex change occurred in 12 cysts, in 7/26 (27%) patients and within 60 days of starting Crizotinib in 10 cysts. Imaging features were falsely concerning for malignancy or abscess in 4/26 patients.

**Conclusion:**

Most CARCs resolve spontaneously, or have a benign evolution despite enlargement and other features concerning for malignancy or infection on imaging. This unusual manifestation of chemotherapy should be recognised, particularly by radiologists, so that inappropriate treatment decisions are avoided.

## Background

Anaplastic Lymphoma Kinase (*ALK*) gene rearrangements were first identified in NSCLC in 2007 [1] and subsequently found to be present in approximately 4% of patients with NSCLC [2]. Crizotinib (Xalkori, Pfizer; PF0234066), a tyrosine kinase inhibitor with efficacy against ALK [3], c-MET [4] and ROS1 [5] kinase, has demonstrated superior efficacy over chemotherapy in the first line systemic treatment of advanced ALK-rearranged NSCLC and represents standard of care for this patient population [6]. With observed median progression free survival (PFS) of 10.9 months and further benefit gained in some cases by continuing crizotinib beyond progression [7], many patients remain on this treatment for years. CT scans performed regularly to monitor disease response also reveal crizotinib related toxicities. Complex renal cysts were reported as a rare complication of crizotinib therapy during clinical trials, initially with an incidence of 4% [8], and more recently, of up to 22% [9, 10]. These cysts have even been noted to invade extrarenal spaces and in a minority, these complex masses can be symptomatic with flank pain [10]. Complex renal lesions arising while on crizotinib therapy can regress without intervention, regardless of whether crizotinib therapy is ceased [9, 11]. We performed a retrospective analysis of the incidence and pattern of evolution of all renal lesions in CT in lung cancer patients who received crizotinib at our centre.

## Methods

Patients with advanced *ALK*-rearranged NSCLC who had been prescribed crizotinib at our institution were identified via the institutional Thoracic Malignancies Cohort database that commenced data collection in July 2012. The cohort included patients on clinical trial and those receiving crizotinib via a special access scheme with Pfizer. The collection of data for our retrospective study was conducted under an ethics approved protocol: the Peter MacCallum Cancer Centre thoracic malignancies cohort study (Peter MacCallum Cancer Centre Human Research Ethics Committee project 11/88) to which patients gave consent to allowing their clinical data and tissue biopsies to be used for future research. To be included in our study, patients needed to have been recruited to the cohort prior to July 2014, have been on crizotinib therapy for at least 2 months, had intravenous contrast enhanced Computed Tomography (IVCECT) prior to commencement of crizotinib therapy and regular follow up IVCECTs, with all scans covering the renal region completely. Patients whose baseline or most follow up CT scans were suboptimal (from lack of IV contrast or movement artefacts affecting assessment of kidneys), those who had proven renal metastases at baseline and those who had interventional procedures on kidneys other than biopsy while on crizotinib were excluded from this analysis. Patients whose baseline scan was ^18^FDG PET/CT scan without IV contrast were included in our study only if the CT component of the PET scan unequivocally showed the renal lesion identified in the subsequent IVCECT. The presence, size, number and morphology of all renal lesions were analysed at baseline and in all CT scans performed while on crizotinib by three radiologists [DJ, KM and BP], obtaining consensus regarding lesion description and morphology as described later. Whilst the vast majority of scans were performed in-house, with sub-millimetre axial helical CT acquisition [Siemens Definition AS+, Siemens AG, Germany], images of the renal region available for review on the image archiving system were the standard 5 mm thick axial and coronal reconstructions.

Renal lesions were included if they were at least 4 mm and were able to be assessed in at least two consecutive scans. They were considered as cysts if they were well defined and showed fluid attenuation [less than 20 HU]. As the CT scans were performed to stage lung cancer, the renal region was covered in most cases only in the portal venous phase, technically not permitting accurate classification of renal lesions by Bosniak criteria. Renal cysts were considered complex if they demonstrated poorly defined margins, density higher than simple fluid (for lesions larger than 1 cm), septae or solid or mixed cystic and solid appearance. Significant renal cystic change was defined as an increase or decrease in size of the cyst by more than 3 mm, or development of any of the complex features mentioned above. Additional patient, malignancy and treatment related information was extracted from the prospective cohort database and collected on retrospective review of clinical records.

## Results

Thirty five patients with NSCLC received crizotinib therapy at our centre in the defined 4 year period. Nine of these patients were excluded from our study; 6 due to suboptimal imaging, 1 due to biopsy proven renal metastases on baseline scan, 1 due to nephrostomy while on treatment and 1 due to an insufficient period of treatment with crizotinib. Of the remaining 26 patients, 10 (38%) remained on crizotinib at our data collection cut-off and only one patient discontinued therapy due to complex renal cyst formation. Patient, tumour and treatment characteristics are presented in Table 1.

**Table 1:** Patient, tumour and treatment characteristics (*N* = 26)

Age (median, range)	52 (34-66)
Sex (Female)	42%
Ethnicity	Asian 31%, Caucasian 69%
Smoking status	Never 58%, light (<10-pyHx) 15%, heavy (>10pyHx) 27%
Diagnosis	Stage IV 96%, confirmed Adenocarcinoma 96%. ALK FISH rearranged: 92%.
Crizotinib provision	Trial: 69%, Access scheme: 31%
Crizotinib duration (median, range weeks)	58 (22-240)
Crizotinib as line of treatment	1st 34%, 2nd 31%, 3rd 27%, 4th 0%, 5th 8%.
Best radiological response	PD 0%, SD 12%, PR 73%, CR 15%

*PD* progressive disease, *SD* stable disease, *PR* partial response, *CR* complete response

Eleven of 26 (42%) patients had no cysts as per our criteria at baseline or in the study period [8 had no cysts at all; 3 had cysts smaller than 4 mm]. 4 of 26 (15%) had cysts at baseline that remained stable on crizotinib treatment. 1 of these patients had a cyst that was complex (due to high density content) at baseline. The remaining 11 (42%) developed a significant change in renal cysts as per our study criteria in a total of 28 renal lesions.

### Radiologically significant renal cyst changes

**Fig. 1:**
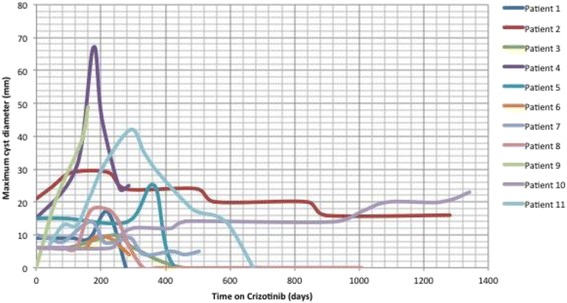
Evolution of CARCs in our cohort: Graph demonstrating evolution in size of the largest CARC in mm (Y-axis) versus days on crizotinib (X-axis) in 11 patients

Eight of the 11 patients with significant change in the renal lesions had more than 1 lesion, with as many as 5 in 1 patient. The evolution in size of the 28 cysts varied greatly between and within patients as demonstrated in Table 2 and Fig. 1. Interestingly, all patients who had more than one cyst showed a mixed pattern of cyst evolution Among them, these 11 patients had 20 cysts at baseline that subsequently showed significant changes; 8 new CARCs were noted in four patients during the study period.

**Table 2:** Cyst evolution in patients with significant renal cystic change (*N* = 11): PR - partial regression

Patient #	Duration of crizotinib (days)	No: of Cysts during study period	Mixed pattern of evolution of lesions	New cysts	Enlarged from baseline			Stable during study period	Regressed from baseline
					Then regressed		Ongoing enlargement		
					CR	PR			
1	280	2	Y	0	1			1	
2	1344	1	N	0		1			
3	678	2	Y	0	1				1; CR
4	126	5	Y	4	(+4)	1			
5	686	4	Y	0	1			2	1; PR
6	150	3	Y	0		1		2	
7	547	1	N	0		1			
8	1109	4	Y	1	2 (+1)			1	
9	160	2	Y	1			(1)	1	
10	1477	1	N	0			1		
11	1456	3	Y	2	1(+2)				
Median age 48 years; 45% F; 64% Asian	Median 678	28		4/11 patients	13/28 cysts	4/28 cysts	2/28 cysts	7/28 cysts	2/28 cysts

*CR* complete regression, (*+*) new lesion

### Size

Nineteen of the 28 lesions in these 11 patients had a significant growth of renal cysts by our definition (Table 2). In these growing lesions, the median maximum size reached was 23 mm (range 9 – 67 mm) after a median of 178 days (160 to 1342) on crizotinib. The median maximum increase in cyst size from baseline was 10 mm (range 4-52 mm) which corresponded to a median percentage increase in size of 63% (range 40 to 347). 95% (18/19) grew by more than 50%. The median rate of growth was 18 mm/year.

**Fig. 2:**
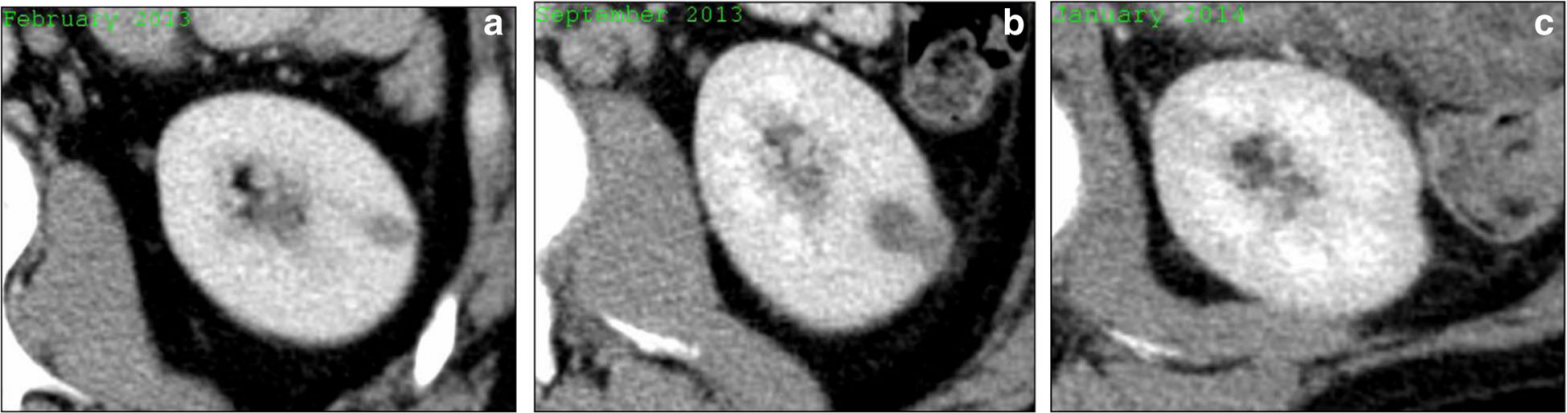
Pattern of evolution of one of the CARCs in patient 1: Axial CT images of the left kidney show enlargement of a 9 mm cyst in patient 1 that was present at baseline in February 2013 (**a**) to 17 mm in September 2013 (**b**) followed by spontaneous resolution leaving a cortical scar in January 2014 (**c**) with ongoing crizotinib therapy

The most common pattern of evolution of the renal cysts was enlargement from baseline followed by spontaneous regression (17/28 lesions), either partially (13/17 lesions) or completely (4/17 lesions), with residual mild cortical scarring (Fig. 2). New cysts developed in 4 patients; 3 of these patients had other pre-existing lesions that were enlarging when new lesions arose. Although *de novo* cysts did arise and significantly evolve, all 11 patients in whom significant renal cystic change occurred had at least 1 renal cyst at baseline. Eleven other patients without any cysts at baseline did not go on to develop renal cysts.

**Fig. 3:**
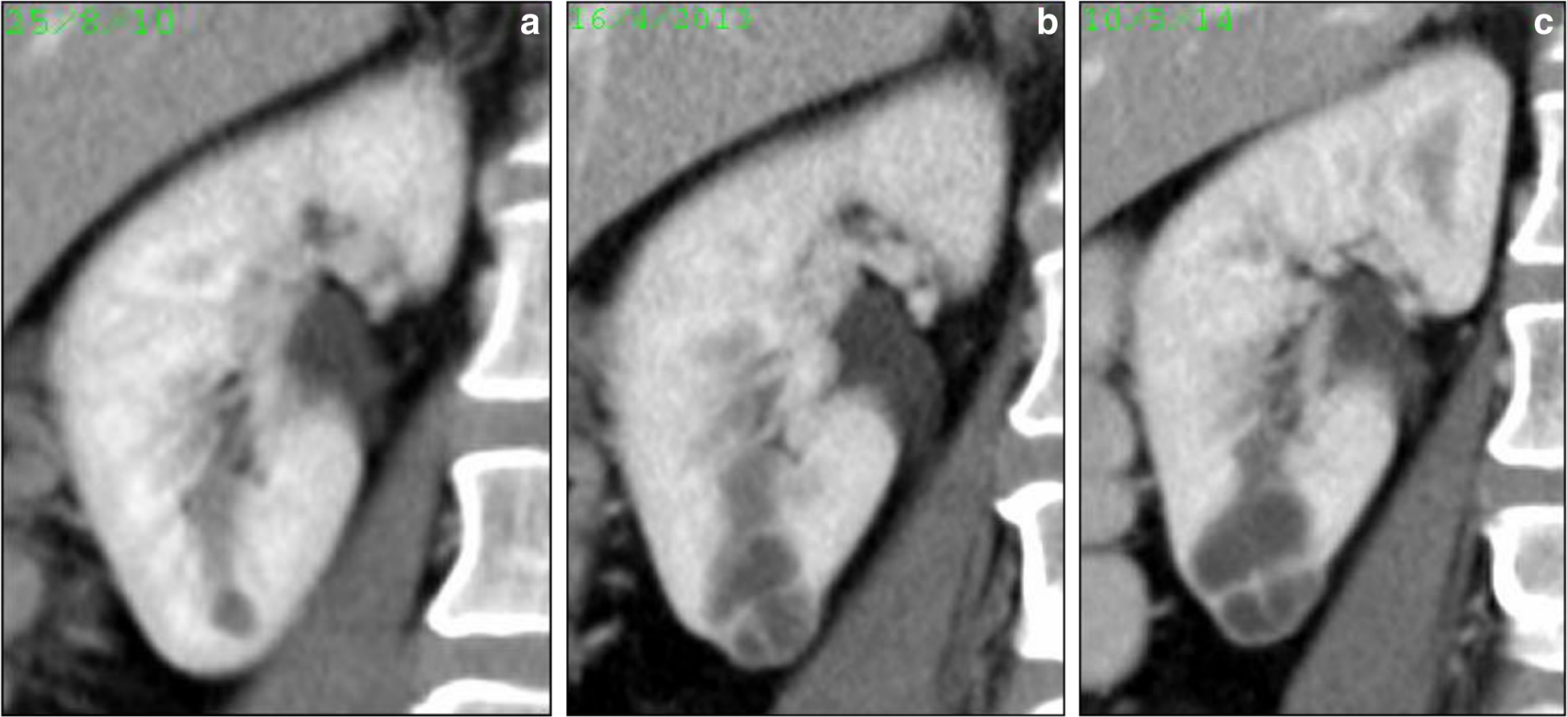
Ongoing enlargement of CARC: Coronal CT images show continued slow enlargement of a right lower pole renal cyst, 6 mm at baseline in July 2010 in patient 10 on crizotinib over 45 months from start of treatment at time points August 2010 (**a**), April 2013 (**b**) and May 2014 (**c**)

Less common patterns of evolution of renal lesions noted concurrently in patients with significantly changing cysts were stable cysts (7 lesions in 5 patients), regression of cysts existing at baseline (2 lesions in 2 patients; 1 with partial and the other with complete regression), and ongoing enlargement. 2 patients showed ongoing enlargement of renal cysts at the end of our study period. 1 patient had a cyst that continued to enlarge at data cut-off, from 6 mm to 27 mm (Fig. 3) over 45 months on treatment. A new cyst that developed in another patient 2 months after start of crizotinib also continued to enlarge, reaching 49 mm on imaging 2 months later, shortly before the patient died due to disease progression.

### Complexity

**Fig. 4:**
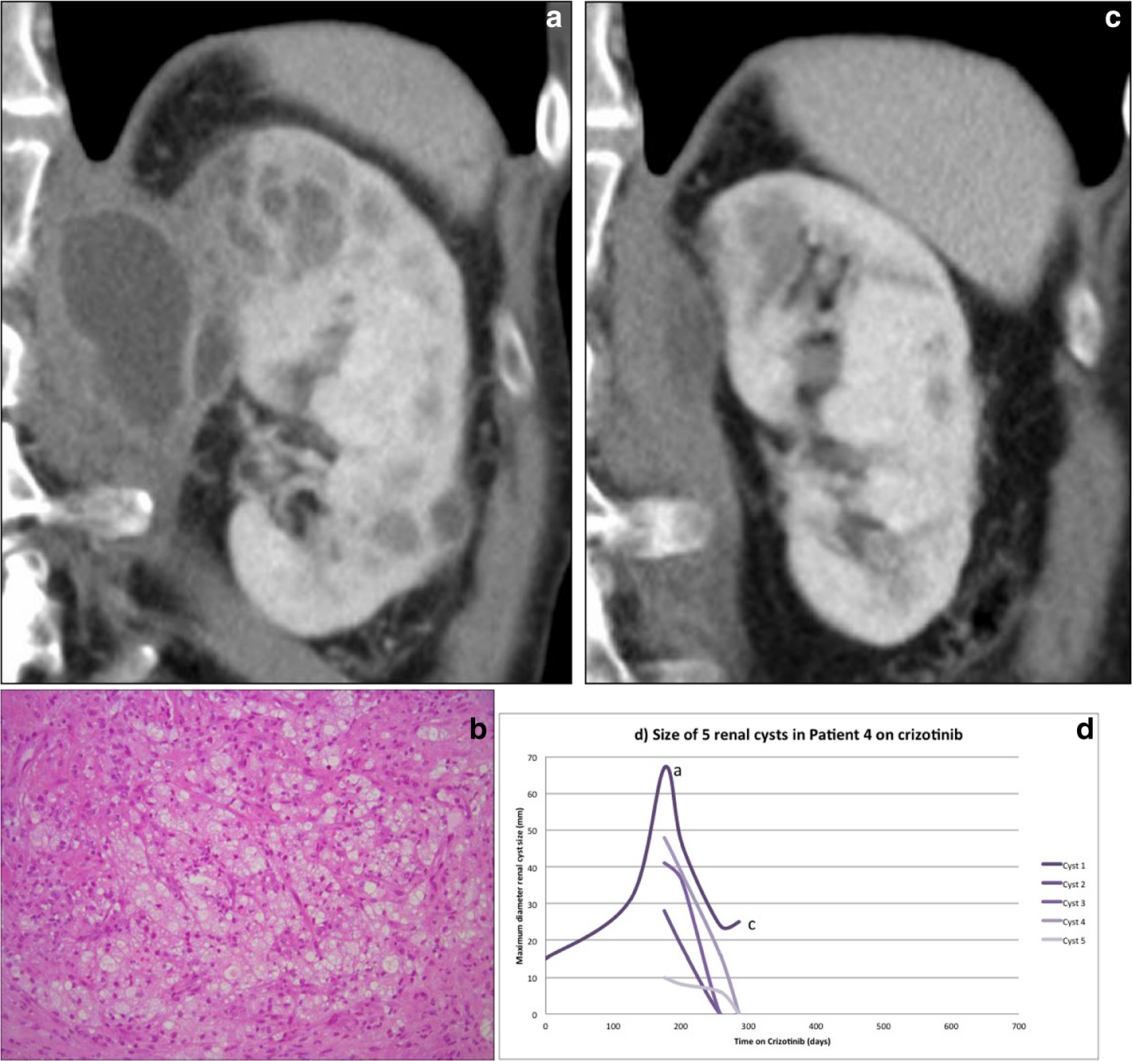
Resolution of CARC upon ceasing crizotinib in patient 4: Coronal CT demonstrating left renal cysts with perinephric and psoas invasion (**a**). Histology of CT guided biopsy from left psoas lesion revealed xanthogranulomatous inflammation (Haematoxylin and eosin, original magnification x200) (**b**). Coronal CT demonstrating resolving cystic changes after discontinuing crizotinib (**c**). Graph: Maximum renal cyst diameter (mm) versus days on crizotinib, demonstrating evolution of five cysts in this patient (**d**)

**Fig. 5:**
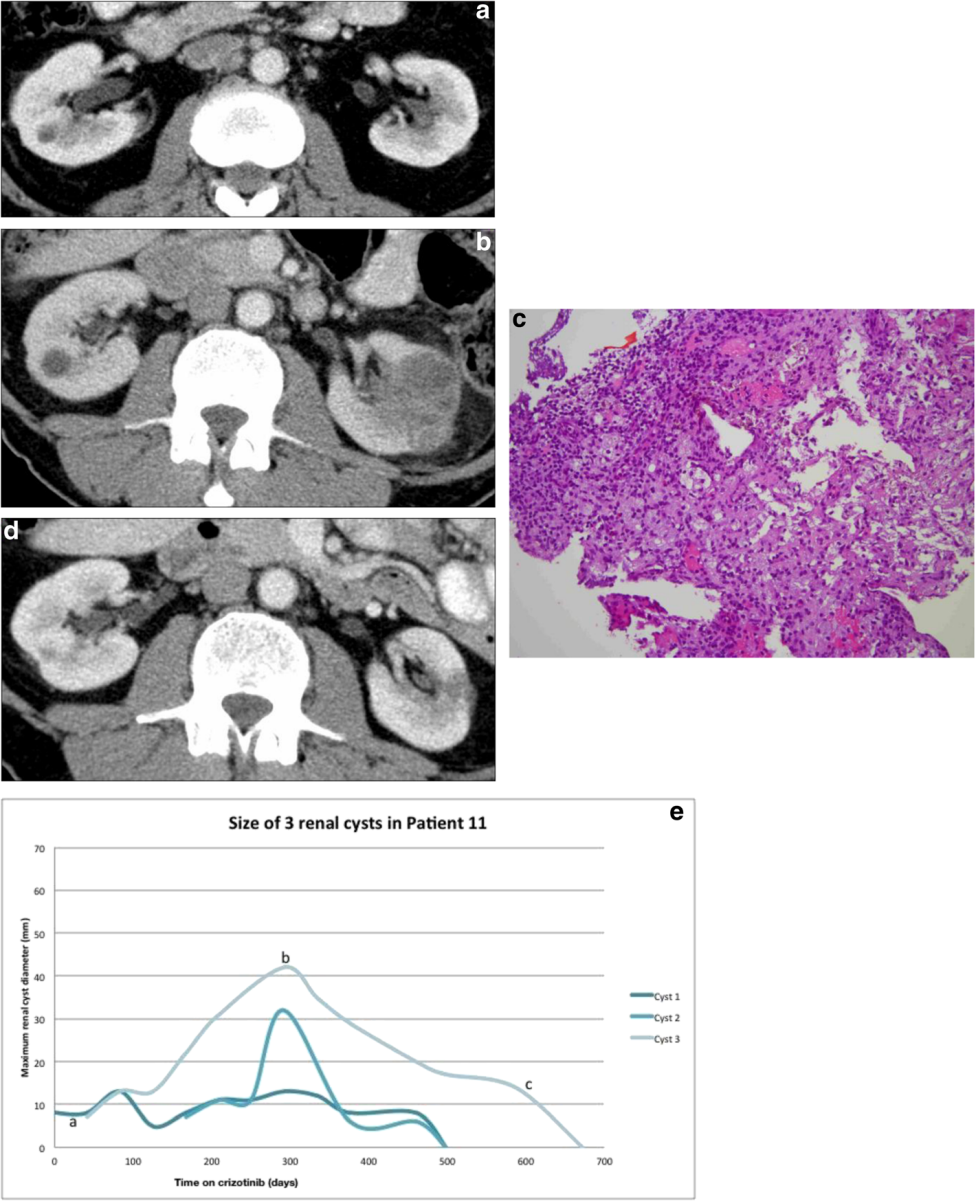
Resolution of CARCs without ceasing crizotinib in patient 11: Baseline scan demonstrated an 8 mm cyst in the right kidney (**a**). Enlarging right renal cyst with no complex features and the new left renal cyst with mixed solid and cystic areas and poorly defined margins (**b**). A new right upper pole cyst with septations is not shown. CT guided aspiration/ biopsy of the left renal lesion revealed xanthogranulomatous inflammation (Haematoxylin and eosin, original magnification x200) (**c**). All three cysts spontaneously resolved with ongoing Crizotinib therapy (**d**). Graph: Maximum renal cyst diameter (mm) versus days on crizotinib, demonstrating evolution of the three cysts in this patient (**e**)

The development of complex features, as defined above, apart from simple changes in size, occurred in 12 cysts, affecting 7/26 (27%) patients overall (Table 3). The median (range) time on crizotinib to development of initial and most complex changes were 172 (0 to 380) days and 199 (130 to 380) days respectively. In 10 cysts, the most complex changes were seen within 60 days of onset. The earliest development of new complex features was seen after 51 days on crizotinib. Bosniak classification was not applied but development of lesions with septations or mixed cystic and solid appearances were noted to be the two most common patterns of complex change in CARCs. Psoas muscle or abdominal wall invasion was seen in 2 lesions in one patient (Table 3). In 4/26 patients, the imaging features of the lesions were concerning for malignant change or abscess and 2 of these patients (Figs. 4 and 5) developed flank pain. Subsequent CT guided biopsy and diagnostic aspiration of few millilitres of cyst contents in these 2 patients (from psoas lesion in one patient and from the renal lesion in the other) revealed benign histology, with both samples showing xanthogranulomatous inflammation. The biopsies showed degenerate cellular debris, fibrosis and a mixed inflammatory infiltrate, including lymphocytes, neutrophils and numerous macrophages, many with foamy cytoplasm. No residual cyst wall was identified, no micro-organisms were seen or cultured, and no malignant cells were present. Both patients had resolution of cystic changes, one after cessation of crizotinib (Fig. 4) and the other despite ongoing treatment with crizotinib (Fig. 5).

**Table 3:** Analysis of complex changes^@^ demonstrated by CARCs

No:	Patient (Ref Table 2)	No: of cysts	Number of cysts demonstrating:						
			Enlargement	New hyper-density	Septation	Mixed cystic and solid appearance	Uniform solid appearance	Poor definition of margins	Psoas/abdominal wall extension
1	#1	1	1	1	-	-	-	-	-
2	#4	5	5	-	4	5	**-**	2	2
3	#5	1	1	1	-	-	1	-	-
4	#8	1	1	1	1	-	-	1	-
5	#9	1	1	1		1	-	-	-
6	#10	1	1	-	1	1	-	-	-
7	#11	2	2	-	1	1	-	1	-
Total	7	12	12	4	7	8	1	4	2

Complex change^@^: does not include lesions with change only in size

### Correlation between evolution of renal cysts and disease response/ renal function

Of the 11 patients with significant renal cystic change, 2 had progressive disease and 9 had continued response (2 complete, 7 partial) at the time of maximum cystic change. There was no apparent association between cyst evolution and renal impairment. The median (range) serum Creatinine was 78 (57 to 92) μmol/L at commencement of Crizotinib and 79 (61 to 106) μmol/L at the time of maximum cystic change. Urinalysis was performed on 5 patients at the time of maximum cystic change and all results were normal.

## Discussion

In the general population, renal cysts are thought to be acquired lesions that evolve from diverticula in distal convoluted and collecting renal tubules [12]. The prevalence of CT detected simple cysts increases with age and has been estimated at 27.5% in 40-60 year olds; 49% in those 60-80 years and 60.6% in those above 80 [13]. The natural history of simple renal cysts is to slowly enlarge, with reported average size increase and rate of enlargement of 1.6 mm and 3.9% per year, respectively [14]. Thus, the prevalence of renal cysts (58%) in our patient cohort (median age 52 years) at baseline was higher than expected.

Within the limits of our small sample size, the rate of growth of renal cysts in our patients on crizotinib was noted to exceed that expected in the natural history of simple cyst evolution. A potential limitation of our study is the lack of comparison in evolution of renal cysts in *ALK*-rearranged NSCLC. There was not an apparent development of renal cysts in *ALK*-rearranged NSCLC patients who received chemotherapy on trial [10] but larger population studies would be difficult to undertake given the efficacy and availability of crizotinib in this population moving forward.

Using predefined criteria for significant change in size and complexity of renal cysts, our study confirms the previously reported increase in size and complexity of renal cysts after commencing crizotinib. In this report we describe the highest incidence of radiologically significant renal cyst change (42%) and new complex features (27%) to date. Our definitions of significant renal cystic change differ only slightly from previous reports. Lin et al. [9] reported a 22% incidence of significant renal cystic change as defined by new renal cysts more than 4 mm in size, enlargement or regression of previous cysts by more than 4 mm or development of complicated renal cysts (Bosniak IIF or higher). Schnell et al. [10] report 2% of cysts to significantly enlarge defined as an increase by more than 50%, however this rate was determined based on cyst detection at the 6 month scan. Our cohort demographics were similar to previous reports in terms of smoking status, adenocarcinoma and median age, reflecting the expected characteristics of patients with NSCLC harbouring *ALK* gene rearrangement. We have demonstrated a variety in the pattern of cyst evolution, even within the same kidney. The clearly dominant pattern of evolution of CARCs in our study was, as reported in literature, asymptomatic cysts that enlarged and spontaneously regressed without discontinuation of crizotinib or need for intervention. It did appear that cysts regressed slightly faster in our single case in whom crizotinib was discontinued (Fig. 4) than in those who continued on treatment (Fig. 5). In addition to this dominant pattern of evolution of renal cysts, we also noted three other previously unreported patterns, namely stability of cysts detected at baseline, regression of lesions from baseline and progressive enlargement of lesions from baseline. The pattern of ongoing cyst enlargement we noted, however, likely represents only a rarer pattern of very slow spontaneous regression, as one of the 2 patients in whom this pattern was observed started showing spontaneous regression of his CARC 16 months after the end of our study period and the other patient died due to disease progression after 5 months of crizotinib therapy.

Renal cysts can be graded using the Bosniak classification system that scores the cyst complexity from I-IV [15]. Higher grades have a higher likelihood of malignancy and current recommendations for Bosniak class III/IV renal cysts include surgical resection [16]. Accurate assessment of cyst morphology and enhancement characteristics requires both non-enhanced and IV contrast enhanced scans to be performed [17]. CT scans in our study were routinely performed only in the portal venous phase. This limitation of our study was addressed by applying commonly accepted features of complexity in renal cysts to the portal venous phase scans. The two commonest complex features we noted in CARCs were the development of multiple septations and a mixed cystic and solid appearance.

The differential of renal cysts demonstrating complex features in a patient with NSCLC includes infection, renal metastases and primary renal malignancy. In two of our patients with CARCs, the imaging and clinical features were concerning enough to warrant a biopsy. In both, CT guided aspiration and biopsy revealed benign histology of xanthogranulomatous inflammation with negative bacterial culture and no malignant cells evident. Several recent case reports have also documented benign pathology upon aspiration of complex cysts in patients with NSCLC treated with Crizotinib [18–20]. Prior knowledge and prompt recognition of CARCs may help avoid unnecessary biopsy/drainage and suboptimal treatment of lung cancer.

While reported regression of the CARCs may have been hastened by drainage [18–20], our experience suggests that these lesions spontaneously resolve.

Percutaneous drainage may be warranted to relieve symptoms of enlarging cysts or to investigate for infection if concerning clinical features such as fever are apparent. We propose that, when found incidentally on imaging, CARCs could simply be observed without intervention or cessation of crizotinib.

The pathogenesis of renal cyst development due to crizotinib is unknown. In response to acquired resistance to crizotinib, several more potent next generation ALK inhibitors have been developed and are now used in the clinical setting [21] Renal cysts have not been reported as a complication of these more specific ALK directed therapies indicating that cystogenesis may be driven by inhibition of other molecules targeted by crizotinib. Crizotinib was initially developed as a c-MET inhibitor. The ligand to c-MET, hepatocyte growth factor (HGF) is thought to mediate human renal cyst formation [22]. Thus it would be paradoxical that inhibition of c-MET by crizotinib would drive cyst formation unless an unidentified feedback mechanism increased levels of HGF to drive cyst formation via another target.

Pre-clinical studies investigating an apparent link between testosterone and polycystic kidney disease have conflicting results [23, 24] and although crizotinib has been shown to reduce testosterone levels [25] we were not able to investigate testosterone levels in our retrospective analysis. Although small numbers don’t allow statistical certainty, it did appear that renal cysts developed in a higher proportion of Asian patients as previously reported [9].

## Conclusions

In summary, development of complex renal cysts is not an uncommon side effect of treatment with crizotinib. Cysts are typically benign, asymptomatic and resolve spontaneously on continued crizotinib therapy. As the use of crizotinib becomes more common, it is imperative that radiologists and treating physicians are aware of CARCs and their temporal evolution.

## Abbreviations

ALK: Anaplastic Lymphoma Kinase
ALK-I: Anaplastic Lymphoma Kinase inhibitors
CARC: Crizotinib Associated Renal Cyst
CT: Computerised tomography
IVCECT: Intravenous contrast enhanced Computed Tomography
NSCLC: Non small cell lung cancer
PFS: Progression free survival
